# Psychosocial and Physiologic Characteristics of Patients with Non-epileptic Events: A Retrospective Study

**DOI:** 10.7759/cureus.6767

**Published:** 2020-01-24

**Authors:** Dipali Nemade, Vikram Shivkumar, Paul Ferguson, Jaysingh Singh, Sona Shah

**Affiliations:** 1 Neurology, Marshall University School of Medicine, Huntington, USA; 2 Neurology, Ohio State University, Huntington, USA

**Keywords:** conversion disorder, non-epileptic seizures, psychogenic non-epileptic seizure (pnes), video electroencephalography (veeg)

## Abstract

Background

The main focus of this study is to aid early identification of psychogenic non-epileptic seizure (PNES) patients by identifying physical and psychosocial characteristics to reduce the health care burden, to reduce the unnecessary use of anti-epileptic medications and their side effects, and maximizing cost-effective use of video electroencephalography (VEEG).

Methods

We analyzed PNES subject data from VEEG monitoring performed at the Epilepsy Center at the Marshall University School of Medicine. We reviewed more than 360 episodes in 54 subjects older than 18 years (mean age ± standard deviation (SD): 48 ± 12.97 years; 83% female).

Results

We found that most of our PNES patients were older than 45 years of age (66.7%), females (83.3%); obese (66.6%) or overweight (18.5%); either single (33.3%), separated (7.4%), divorced (22.2%), or widowed (14.8%); of low education, unemployed (either received government assistance (83.3%) or disability benefits (57.4%)) with associated physical illness (85.2%) and psychiatric illness (96.3%).

Conclusion

Our study adds to the current knowledge of the sociodemographic and sociocultural variability of PNES. It might enable early diagnosis and management of patients with PNES.

## Introduction

A psychogenic non-epileptic seizure (PNES) is defined by an absence of electroencephalographic (EEG) changes in association with behavioral manifestations that correspond to the clinical phenomena witnessed by a family member or friend during prior events [1-2]. PNES is a common phenomenon that contributes to as much as 40% of video electroencephalography (VEEG) characterization in specialty epilepsy centers with an estimated cost ranging in excess of 650 million dollars annually [3]. The diagnosis of PNES can often be delayed by failure to consider the events as non-ictal in nature. This can lead to the inappropriate use of anti-epileptic drugs with possible untoward side effects, in addition to the socioeconomic impact of an inappropriate diagnosis [4]. Failure to diagnose PNES leads to frequent emergency department (ED) visits and subsequent hospitalizations leading to increased health care expenditures.

There is insufficient data about the effect of sociodemographic variables on the incidence of PNES. Some studies have reported a higher incidence in females and lower education groups, while others have not found such differences [1, 5]. Our study aimed to determine whether there was any correlation between sociodemographic factors and psychological comorbidities in adults with PNES diagnosed by VEEG monitoring at our tertiary referral center. These results might enable health care personnel to identify PNES patients earlier by recognizing common phenotypes and contributing psychosocial and physiologic characteristics. This will lead to early diagnosis of PNES which will lead to a reduction in health care expenditures, as well as reduce inappropriate use of anti-epileptic medications.

## Materials and methods

Standard protocol approvals, registrations, and patient consents

This study is a retrospective chart review conducted in the Department of Neurology at Marshall University School of Medicine in Huntington, West Virginia. The study’s research protocol was approved by the Marshall University Institutional Review Board and Hospital Ethics Committee prior to study implementation (#975386-1).

Diagnosis of PNES

A diagnosis of PNES was made by an epileptologist after capturing, on VEEG, at least one of the patient's typical events as indicated by the patient or family member. VEEGs were reviewed based on the American Clinical Neurophysiology Society (ACNS) guidelines with and without filtering, and various average and bipolar montages were used to identify ictal EEG rhythms. A PNES event was defined by an absence of EEG changes in association with behavioral manifestations and correspondence of the clinical phenomena with a previous event witnessed or identified by a family member or friend [1-2].

Recruitment of cases

PNES patients diagnosed by VEEG from August 2013 to July 2016 (n = 54) were selected from Marshall Health medical records. An International Classification of Diseases, 10th Revision (ICD-10) search was conducted to identify patients who have been diagnosed with PNES. Information regarding sociodemographic variables, such as age, sex, race, body mass index (BMI), marital status, education, occupation, disability, alcohol, tobacco and drug abuse, age of onset of seizures, number of years with seizures and frequency of seizures, associated physical and psychiatric illness, type of insurance, and VEEG results, was collected from the patients' records. 

For our study, associated psychiatric illness was defined as being currently treated with Federal Drug Administration (FDA)-approved medications, including antidepressants, anxiolytics, mood-stabilizing anti-epileptics, lithium, antipsychotics, or if patients were diagnosed by a psychiatrist as having any of the following conditions: depression, anxiety, bipolar disorder, post-traumatic stress disorder, dissociative disorder, borderline personality disorder, or pseudocyesis. Associated physical illness included a diagnosis of migraines, stroke, cardiovascular disease, high cholesterol, hypertension, diabetes, hypothyroidism, asthma, gastroesophageal reflux disease (GERD), irritable bowel syndrome (IBS), cancer, arthritis, chronic pain (refractory back pain and pain syndromes, such as fibromyalgia), and insomnia. 

Inclusion and exclusion criteria

Inclusion Criteria

a. Age equal to or more than 18 years old.

a. Must have a VEEG confirmed diagnosis of only PNES.

Exclusion Criteria

a. Patients ages < 18 years old were excluded from the study.

b. Patients with a combination of epilepsy and PNES were excluded.

Data analysis and availability

The data was compiled and analyzed using frequency and percentage using statistical software IBM Statistical Package for Social Sciences (SPSS), version 23 (IBM SPSS Statistics, Armonk, NY). The de-identified data not presented in the article will be made available to investigators by request.

## Results

From August 2013 to July 2016, we reviewed 614 patients who had a seizure disorder, out of which 54 patients were given a diagnosis of PNES as per the ICD-10 classification. Some variables, such as a history of physical and sexual abuse and suicidal ideation, have been shown to be risk factors for PNES in prior studies [6-7]. However, on review of our records, it was found that most patients did not answer these questions on the intake form, and hence, these variables were excluded. We reviewed more than 360 episodes in 54 subjects older than 18 years (mean age ± standard deviation (SD): 48 ± 12.97 years; females: 45; males: 9). The physical characteristics of the patients are shown in Table 1.

**Table 1:** Physical Characteristics of Patients with Psychogenic Non-epileptic Seizures (n = 54) BMI: body mass index

Physical characteristics		N	%
Age	18 - 45 years	18	33.3
	More than 45 years	36	66.7
Sex	Males	9	16.7
	Females	45	83.3
Race	Caucasian	53	98.1
	Hispanic	1	1.9
BMI	Underweight (< 18.5 kg/m^2^)	3	5.6
	Normal (18.5 to 24.9 kg/m^2^)	5	9.3
	Overweight (25 to 29.9 kg/m^2^)	10	18.5
	Obese (> 30 kg/m^2^)	36	66.6
History of head injury	Yes	9	16.7
	No	45	83.3

Out of 54 patients, 53 were Caucasian and only one patient was Hispanic. Most of the patients with PNES were either obese (36, 66.6%) or overweight (10, 18.5%); only five (9.3%) had a normal BMI and three (5.6%) were underweight. Nine patients (16.7%) with PNES had a history of head injury. The psychosocial characteristics of the patients are shown in Table 2. PNES patients were either single (18 patients, 33.3%), separated (four patients, 7.4%), divorced (12 patients, 22.2%), or widowed (eight patients, 14.8%). Out of 54, only five patients achieved the highest level of education, i.e., two patients (3.7%) graduated from college and three (5.6%) were undergraduates. Forty-six (85.2%) of them had education between sixth to 12th grade and three (5.6%) were noted to have education below the fifth grade.

**Table 2:** Psychosocial Characteristics of Patients with Psychogenic Non-epileptic Seizures (n = 54) K: kindergarten

Psychosocial Characteristics		N	%
Marital status	Single	18	33.3
	Married	11	20.4
	Separated	4	7.4
	Divorced	12	22.2
	Widowed	8	14.8
	Other	1	1.9
Education	Elementary school (K - 5th grade)	3	5.6
	Middle school (6th - 8^th^ grade)	21	38.9
	High school (9th - 12^th^ grade)	25	46.3
	Undergraduate	3	5.6
	College graduate	2	3.7
Occupation	Unemployed	49	90.7
	Employed	5	9.3
Disability benefits	Yes	31	57.4
	No	23	42.6
Current smoker	Yes	37	68.5
	No	17	31.5
Current alcohol use	Yes	33	61.1
	No	21	38.9
Current illicit drug use	Yes	10	18.5
	No	44	81.5
Insurance	Medicare/Medicaid	45	83.3
	Private/commercial	9	16.7
Associated physical illness	Yes	46	85.2
	No	8	14.8
Associated psychiatric illness	Yes	52	96.3
	No	2	3.7

Forty-nine patients (90.7%) were unemployed, 45 (83.3%) of them were on government assistance insurance, and 31 (57.4%) received disability benefits. Forty-six (85.2%) and 52 patients (96.3%) had associated physical and psychiatric illnesses, respectively. The mean number of seizures per patient was 4.7 (range: 1 - 14) with 44 patients (81.5%) having one or more seizures per month. Forty-three patients (79.6%) had the onset of seizures after the age of 20, while 42 patients (77.8%) had seizures for less than 10 years.

## Discussion

In our present retrospective study, the mean age ± SD for PNES patients was 48 ± 12.97 years (range: 20 - 71 years), which was similar to a study conducted by Hill et al. in which the mean age ± SD was 39.4 ± 12.8 years [8]. PNES has a female predominance ranging from 66 to 99% in various studies. In our study, we found a higher female (F = 45) prevalence as compared to males (M = 9) which is consistent with female preponderance narrated in conversion disorder, one of the salient psychiatric conditions underlying PNES [9-10].

In our study, there were 36 obese patients (66.7%) and 10 overweight patients (18.5%), which is consistent with a study by Elliott et al. [11]. This is attributed to somatic manifestations of psychological distress. It has been proven by various studies that weight problems are more common in patients with psychiatric illness [12]. We observed that PNES patients admitted for VEEG monitoring had larger body habitus as compared to epilepsy patients.

The majority of PNES patients (18, 33.3%) were either single, separated (four, 7.4%), divorced (12, 22.2%), or widowed (eight, 14.8%), and one patient (1.9%) did not disclose their marital status. This may be due to the lack of a social support system. Few studies have shown that married individuals are more likely to enjoy better mental health [13-14]. Some literature did reveal an association between dysfunctional family relationships and PNES [15-17]. However, a few other studies have not shown any link between marital status and PNES [18-19].

It is already known that the diagnosis of PNES is linked to various psychiatric diseases and symptoms. However, these conditions are also seen in patients with epilepsy and are not specific to PNES. Psychiatric conditions are often not known at the time of presentation and may instead be revealed later as part of the evaluation [18, 20-21]. A study including more than 250 subjects narrated that PNES patients were significantly more likely than those with epilepsy (66% vs 27%, respectively) to have one or more diagnoses, including fibromyalgia, systemic exertion intolerance disease, chronic pain syndrome, headaches, irritable bowel syndrome (IBS), asthma, and gastroesophageal reflux disease (GERD) [22]. This is consistent with our study where 52 (96.3%) and 46 (85.2%) PNES patients had some sort of psychiatric and physical illness, respectively, as defined above. 

Only five patients achieved the highest level of education, i.e., two patients (3.7%) graduated from college and three (5.6%) were undergraduates. The remaining 46 patients (85.2%) had 6th to 12th-grade education and three (5.6%) were noted to have education at or below the 5th grade. This is in contrast to other studies in which they did not find the influence of education on the prevalence of PNES [18-19, 23]. This study showed that 49 (90.7%) out of 54 PNES patients were unemployed and 31 (57.4%) out of 54 patients received disability benefits. This is also revealed in studies by Reuber et al. where 56% of patients continued to depend on state-supported financial benefits four years after PNES diagnosis [24-25]. This was also compatible with an article by Walczak et al. where they followed PNES patients for 27 months and the occupational status did not change in 75% of the patients [26].

In conclusion, some risk factors, such as the longer duration of symptoms, older age at onset, lower educational level, limited family support or family conflict, dependent lifestyle, and severe underlying psychiatric disorder (especially somatization or dissociative symptoms), were inconsistently associated with a worse prognosis [27-29]. A limitation of this study is that we did not compare patients with PNES and those with PNES, plus epilepsy. 

## Conclusions

In this current study, most of the patients with PNES were older, female, obese, either single, separated or divorced, of low education, and unemployed (either receiving government assistance or disability benefits) with associated physical and psychiatric illness. Our study adds to the current knowledge of the socio-demographic and socio-cultural variability of PNES. The presence of some of the physiologic and psychosocial characteristics mentioned above might enable earlier identification of PNES. Future studies comparing patients with PNES alone vs PNES patients with epilepsy might further establish the association of PNES with specific psychosocial characteristics. Collaboration between psychiatrists, psychotherapists, and neurologists is essential for the early diagnosis and management of PNES.
